# Poor Transferability of Species Distribution Models for a Pelagic Predator, the Grey Petrel, Indicates Contrasting Habitat Preferences across Ocean Basins

**DOI:** 10.1371/journal.pone.0120014

**Published:** 2015-03-06

**Authors:** Leigh G. Torres, Philip J. H. Sutton, David R. Thompson, Karine Delord, Henri Weimerskirch, Paul M. Sagar, Erica Sommer, Ben J. Dilley, Peter G. Ryan, Richard A. Phillips

**Affiliations:** 1 Marine Mammal Institute, Department of Fisheries and Wildlife, Oregon State University, Newport, Oregon, United States of America; 2 National Institute of Water and Atmospheric Research Ltd., Hataitai, Wellington, New Zealand; 3 Centre d’Etudes Biologiques de Chizé,-CNRS UPR 1934, Villiers en Bois, France; 4 National Institute of Water and Atmospheric Research Ltd., Riccarton, Christchurch, New Zealand; 5 Denny Ecology, Cambridge, United Kingdom; 6 Percy FitzPatrick Institute, DST/NRF Centre of Excellence, University of Cape Town, Rondebosch, South Africa; 7 British Antarctic Survey, Natural Environment Research Council, High Cross, Madingley Road, Cambridge, United Kingdom; University of Lleida, SPAIN

## Abstract

Species distribution models (SDMs) are increasingly applied in conservation management to predict suitable habitat for poorly known populations. High predictive performance of SDMs is evident in validations performed within the model calibration area (interpolation), but few studies have assessed SDM transferability to novel areas (extrapolation), particularly across large spatial scales or pelagic ecosystems. We performed rigorous SDM validation tests on distribution data from three populations of a long-ranging marine predator, the grey petrel *Procellaria cinerea*, to assess model transferability across the Southern Hemisphere (25-65°S). Oceanographic data were combined with tracks of grey petrels from two remote sub-Antarctic islands (Antipodes and Kerguelen) using boosted regression trees to generate three SDMs: one for each island population, and a combined model. The predictive performance of these models was assessed using withheld tracking data from within the model calibration areas (interpolation), and from a third population, Marion Island (extrapolation). Predictive performance was assessed using k-fold cross validation and point biserial correlation. The two population-specific SDMs included the same predictor variables and suggested birds responded to the same broad-scale oceanographic influences. However, all model validation tests, including of the combined model, determined strong interpolation but weak extrapolation capabilities. These results indicate that habitat use reflects both its availability and bird preferences, such that the realized distribution patterns differ for each population. The spatial predictions by the three SDMs were compared with tracking data and fishing effort to demonstrate the conservation pitfalls of extrapolating SDMs outside calibration regions. This exercise revealed that SDM predictions would have led to an underestimate of overlap with fishing effort and potentially misinformed bycatch mitigation efforts. Although SDMs can elucidate potential distribution patterns relative to large-scale climatic and oceanographic conditions, knowledge of local habitat availability and preferences is necessary to understand and successfully predict region-specific realized distribution patterns.

## Introduction

There are typically two general aims of species distribution models (SDMs): (1) to describe the ecological drivers of distribution patterns, and (2) to make predictions of species distributions. Both aims are important in a conservation framework to anticipate how changing environments may impact a species, and to inform a potential management response [1]. When applied appropriately, SDMs can provide useful ecological insights and strong predictive capability [2]. Therefore, SDMs generated by a variety of modelling techniques have been used in diverse applications across multiple species, ecosystems, and scales e.g., [3–6].

Despite their increasing popularity, the usefulness of SDMs as predictive tools has been questioned due to a tendency for model predictive capacity to be tested within the same spatial and temporal range as the training data (interpolation), rather than an assessment of a model’s transferability to novel regions and time periods (extrapolation) [4,7]. For SDMs to be a practical method for predicting species distributions, it is necessary to quantify their generality with independent data to ensure that predictions accurately reflect species distribution patterns away from the calibration region [8]. Model transferability is particularly important for species of high conservation concern if SDMs are being used to target management interventions.

Testing model transferability is feasible and should be a basic requirement for SDMs [9,10], particularly for models intended for conservation planning, but it is rarely assessed [4,7]. The few studies to assess transferability have generated contrasting results, mostly attributed to variation in scale [3–5,11,12]. Large-scale analyses examine whole-species distributions, whereas fine-scale models examine the responses of individuals to local environmental variability [13]. Despite the tendency and temptation to infer a species’ fundamental or realized niche based on SDMs, statistical models are ill-suited for these purposes because they are unable to account for all biotic and abiotic effects on individual fitness [14,15]. Rather, SDMs are a powerful tool to identify a species’ potential and realized distributions [15]. Potential distribution patterns define where a species could live, and realized distributions reflect where a species actually lives in relation to the habitat that is available. From an evolutionary perspective, the large-scale physical and biological processes that drive potential distributions should be congruent across populations and regions, but at a fine-scale, realized distributions are limited by local conditions. Therefore, fine-scale models with good fit to the training data may work well in adjacent habitats with similar characteristics, but fail when extrapolated to more distant areas where environmental conditions and local processes may be more distinct.

Although SDMs have only recently been applied to marine species, the uptake has been swift, including distribution models for highly mobile and dynamic predators such as seabirds [16]. Miniaturised tracking devices have revealed the distribution patterns of many seabirds within the order Procellariiformes (shearwaters, petrels and albatrosses). This information is critical for a group that includes many species, particularly albatrosses and large petrels, which face major threats from fisheries and habitat change [17,18]. Therefore, SDMs have been applied recently to improve the understanding of procellariiform at-sea distribution patterns and define areas of high risk from fishing and other activities [19–21].

Previous studies have documented associations between the distributions of procellariiform seabirds and oceanographic features at various scales e.g., [19,21,22]. These studies emphasize the need to understand how dynamic oceanographic processes generate and sustain prey aggregations and hence determine predator distribution patterns. SDMs have been used to predict habitat use of several albatrosses and petrels in the same region as the underlying distribution data were collected (interpolation), with apparently high predictive capacity [6,19–21]. Additionally, Louzao et al. [18] demonstrated successful temporal transferability of an SDM for the wandering albatross *Diomedea exulans* across decades. However, to our knowledge, the only previous test of spatial transferability of an SDM for a marine predator was for parapatric penguin populations within the same ocean region [23].

Obtaining representative tracking data for species with wide distributions, particularly those that breed on remote islands, is logistically and financially challenging. A more practical approach to assessing distribution patterns may be to extrapolate a reliable SDM based on tracking data from one or more populations, to other regions where there are no tracked birds. The applicability of this approach is tested here using data from the little studied grey petrel *Procellaria cinerea*. This species breeds during winter at several remote sub-Antarctic islands, with the largest population at Antipodes Island in the south west Pacific Ocean (49°42′S, 178°47′E; ca. 53,000 breeding pairs [24]). Grey petrels also breed on the Kerguelen Islands in the south eastern Indian Ocean (49°28′S, 69°57′E; ca. 3,400 breeding pairs [25]), and the Prince Edward Islands in the south western Indian Ocean (46°38′S, 37°55′E; ca. 5,000 breeding pairs [26]). Although little is known about grey petrel distribution or foraging patterns, cephalopods dominate their diet by frequency of occurrence (86.7%) and mass (70.4%; [27]). The dominant at-sea threat to grey petrels is incidental bycatch in longline fisheries [25,28] and their distribution overlaps with nine different regional fisheries management organizations [29]. This study utilizes tracking data from adults from three populations of grey petrels breeding in different ocean basins to (i) document, for the first time, their distribution during the non-breeding season, (ii) develop SDMs to compare the oceanographic characteristics of the habitats used in different regions, and (iii) test model interpolation and extrapolation capabilities. This study represents the first comprehensive test of SDM transferability in pelagic ecosystems, and adds to a limited number of studies that assess transferability in any type of ecosystem at the scale of thousands of kilometres e.g., [30].

## Materials and Methods

### Tracking datasets

Global Location Sensors (GLS loggers; British Antarctic Survey, Cambridge, UK) were used to record the long-term, broad-scale movements of individual grey petrels from three colonies: Antipodes Island, Kerguelen Island and Marion Island within the Prince Edward Islands. GLS loggers record ambient light, allowing latitude and longitude to be estimated based on thresholds in light curves and time of day [31]. GLS loggers are lightweight with a long battery-life, but only provide two locations a day (at local midday and midnight) and have relatively low spatial accuracy (186 ± SD 114 km; [31]). This level of accuracy is adequate to assess the large-scale movement patterns of seabirds.

Twenty-seven GLS loggers were deployed on grey petrels at Antipodes Island in February 2009, 18 (67%) of which were retrieved in March or April 2010. Thirty GLS loggers were deployed on grey petrels on Kerguelen Island in April 2007 or 2008, and 11 (37%) were retrieved in April of the following years, but two failed to download. Three GLS loggers were deployed on grey petrels on Marion Island in May 2009, with one recovered in May 2010. In May 2013, a further nine GLS loggers were deployed at Marion Island, of which four were recovered in April 2014, but one failed to download. Therefore, five (42%) of 12 devices were recovered at this site. The loggers were models Mk9 (n = 33), Mk13 (n = 1), and Mk7 (n = 5), all of which weighed <2.5 g (<0.2% of mean adult body weight) and were mounted on plastic bands fitted to the tarsus. Light data from retrieved loggers were processed according to Phillips et al. [31] to remove unrealistic locations.

### Ethics statement

All scientific procedures were approved by the relevant authorities: Tagging work at Antipodes Island was conducted under permit issued by the New Zealand Department of Conservation and was approved by the animal ethics committee at the National Institute of Water and Atmospheric Research in New Zealand; work at Kerguelen Island was approved by the ethics committee of the French Polar Institute (IPEV); On Marion Island, research was conducted under permit issued by the South African Department of Environment Affairs, following approval by the University of Cape Town’s Animal Ethics Committee. The duration of handling times of all grey petrels lasted < 10 minutes and great care was taken to minimize stress to the animals during tag deployment and retrieval.

### Data implemented in SDMs

Three SDMs were generated using tracking data recorded during the non-breeding period (October to February): an Antipodes model, a Kerguelen model, and a combined model including data from both populations. As in previous studies that generated seabird SDMs using tracking data e.g., [18,20,21], it was assumed that the pooled sample of tracked individual grey petrels was representative of the distribution patterns of the respective population. Due to the small sample of birds tracked from Marion Island, a SDM was not created for this population.

Density contours of 50% and 90% for each population were calculated for each month during the non-breeding phase using a 200-km search radius. Presence data implemented in the three models were the GLS locations within the 50% density contour for each month. In seabird studies, core habitat is commonly defined as the area within the 50% density contour [32]. Pseudo-absence data for each month were uniformly spaced points (every 100 km^2^) within the contiguous 90% density contour of all non-breeding locations (October to February) for each population, including throughout the 50% density polygon. These pseudo-absence data are essentially background data that characterize the habitat available to the petrels. This method enabled a comparison between the core habitat used by petrels (monthly 50% density contour) relative to the habitat available within the general region that the birds could have exploited (90% density contour). No standard method has been developed for implementation of marine megafauna tracking data, which inherently consists of presence-only data, into habitat models. The approach used here allowed background data to overlap spatially and environmentally with presence data, generating presence versus availability models rather than presence versus absence models [33]. Preliminary models tested various methods of generating pseudo-absences, including the use of absence data only from outside the 50% contour (a presence/absence model). This initial effort determined no significant differences in model results or conclusions. Therefore, the presence/availability model approach was applied because it provided a more rigorous test of habitat use patterns by accounting for variation in the availability of habitat throughout the non-breeding range. For both populations, 20% of the presence data and 100 pseudo-absences were randomly subsampled and withheld from each month for model validation. These withheld data were also used for validating the combined model.

### Environmental data implemented in SDMs

A range of environmental datasets were included in models to characterise grey petrel habitat use. These included five static layers: depth (m; General Bathymetric Chart of the World (http://www.gebco.net/), sea bed slope (degrees), month (categorical), geomorphology class (categorical; shelf, slope, rise, plain, valley, trench, trough, basin, hills(s), mountains(s), ridges(s), plateau, seamount; [34]), and distance from seamount (m; [35]). Month was included as a factor in the models to evaluate possible temporal shifts in habitat use. In addition, dynamic environmental variables were derived from oceanographic climatologies and remotely-sensed data. Mixed layer depth (m), surface currents (m.s^-1^), temperature (°C) at 0 m, 10 m, and 50 m, mean temperature at 0–10 m and at 0–50 m were extracted from the CSIRO Atlas of Regional Seas (CARS) [36]. Eddy kinetic energy ((cm.s^-1^)^2^), a measure of variability in surface currents (derived from AVISO: http://www.aviso.oceanobs.com) and surface chlorophyll *a* (mg.m^-3^; derived from SeaWifs images: http://oceandata.sci.gsfc.nasa.gov/) were also obtained. These dynamic oceanographic data describe averaged conditions at a location and month, based on long-term data collection. Environmental values were extracted from each dataset at all presence and background locations, and associated month for dynamic layers (i.e., November presence/background points sampled the November climatologies). All environmental variables included in the SDMs had a spatial resolution of 0.5 degrees, and the dynamic variables had a monthly temporal resolution. For all SDMs, presence and background data from all months were combined into a single model.

### Modelling methods

Response and predictor variables were related in a boosted regression tree (BRT) modelling framework, a method that is able to interpret complex relationships between species and the environment [37]. The first of two algorithms implemented in BRT modelling partitions observations into groups with similar characteristics using regression or classification trees. Boosting is the second algorithm and stems from machine learning where trees are fitted iteratively, emphasizing observations that poorly fit the existing collection of trees [38]. Boosting combines these trees to minimize misclassification errors and improve predictive performance over a single tree model [37]. Boosting is optimized by the learning rate (lr) that sets the weight applied to individual trees, tree complexity (tc) that indicates the number of interactions between predictor variables, and the number of trees (nt) used. Model fit and predictive performance are balanced to reduce overfitting by jointly optimizing nt, lr, and tc with respect to model validation metrics (see below) [39]. Models were tested with specified tc between 1 and 4, and a variable lr (minimum 0.05) that allowed nt to exceed 1000, as recommended by Elith et al. [39]. The advantage of BRT modelling in ecological studies, such as the work presented here, is that it copes with non-linear relationships, and correlated and interacting variables.

The relative importance of a predictor variable in BRT models is determined by its contribution to the model as measured by the number of times it is selected for tree splitting. Predictor variables were removed from models if they contributed less than 5% [40], therefore limiting the chances of overfitting models to the data. Fitted functions are produced by the BRT that show the effect of a focal predictor on the response while controlling for the average effect of all other variables in the model [39]. Species distribution BRT models are effective for understanding the ecological drivers of distribution patterns and are a reliable approach to predict species distributions across multiple scales and species [34,37,41]. When ten different approaches to generating SDMs were evaluated, those based on BRTs had generally high model transferability between regions [42].

As twice the number of grey petrels was tracked from Antipodes Island compared to Kerguelen Island, presence and background locations for the Antipodes population were down-weighted in the combined model to balance the contribution from each population. The combined model of grey petrel distribution was generated using the predictor variables common to both population models to maximize generality. The best models for each population were selected based on two internal performance metrics: (1) The cross-validated area under the receiver operating curve (AUC), which measures the model’s ability to discriminate between used and available sites. AUC ranges from 0 to 1 (1 = perfect discrimination, > 0.7 is considered a “useful model”, [43]); (2) The percent deviance explained (dev) is estimated by the cross-validation procedure and provides a measure of the goodness-of-fit between predicted and raw values to indicate how well the model predicts withheld data [39].

### Spatial predictions and validation of SDMs

Spatial predictions based on the three BRT models were generated across the entire Southern Hemisphere from 25 to 65°S for each non-breeding month (October to February). These maps allowed visual assessment of predicted monthly habitat suitability for grey petrels and a comparison with actual distribution data (monthly 50% and 90% density contours from each population). Additionally, to visually assess extrapolation ability, habitat suitability predicted by each of the three models was compared with the kernel density distribution of grey petrels tracked from Marion Island.

The BRT models for Antipodes and Kerguelen were validated externally using withheld data to test interpolation (within population) and extrapolation (to the other population) capability. The interpolation capability of the combined model was also tested using withheld data from both populations. The transferability of all three models was tested using the track data from grey petrels at Marion Island. Similar to the methods used to designate presence data for the BRT models, all GLS locations of birds tracked from Marion Island that fell within the 50% density contour were considered presence locations, and 435 uniformly spaced points (every 100 km^2^) within the 90% density contour were used as background locations. Habitat characteristics at each presence and background location for the birds from Marion Island were extracted from the same environmental layers described above, at the same spatial and temporal resolutions.

Due to the presence/availability design of the BRT models, k-fold cross validation was applied in the external validation process to assess the predictive capacity of ‘used’ locations [33]. Traditional metrics of model performance, such as AUC, are limited to presence/absence models where predictions are scored as used or unused. With presence/availability models, validation of absence locations are uncertain [33]. The k-fold cross validation binned the predicted habitat suitability of each presence and absence location into equal-interval groups (0 to 1) and the proportion of presence locations in each bin was determined. A Spearman-rank correlation (r_s_) was calculated between bin rank and the proportion of presence locations to assess whether the latter increased with increasing suitability of predicted habitat, indicating good predictive performance [33]. Additionally, we assessed model calibration for the three SDMs to determine whether predictions were proportional to conditional probability of habitat suitability [44]. We generated calibration plots and calculated the point biserial correlation (COR; [30]) using the withheld data from each model to evaluate interpolation, and the presence/background data from Marion Island to evaluate extrapolation.

### Programming and analysis environments

Geospatial processing and analyses were conducted using ArcGIS v10.0 (ESRI, CA, USA), Geospatial Modelling Environment (http://www.spatialecology.com/gme/), Matlab v R2013a (MathWorks, MA, USA), and R [45] using the packages Raster, gbm (1.6–3.1; [46]), and PresenceAbsence, and custom code provided by Elith et al. [39] and Phillips and Elith [44].

## Results

### GLS data implemented in SDMs and validation tests

**Fig 1:**
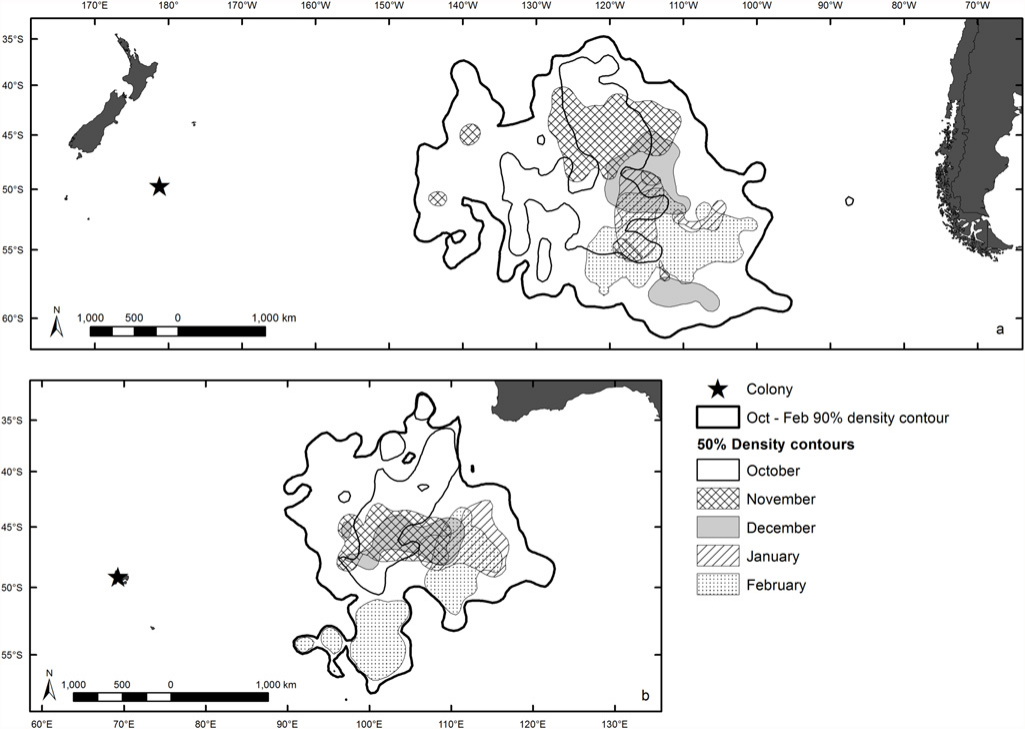
Density contours for tracked grey petrels from (a) Antipodes Island, and (b) Kerguelen Islands. The 90% density contour (thick black line) is derived from all GLS locations during the non-breeding season (October to February) for each population. The 50% density contours were derived for each month of the non-breeding season for each population. Maps in Mercator projection, datum wgs84.

After filtering, 4801 and 2003 GLS locations were available for grey petrels from the Antipodes and Kerguelen populations, respectively. All tracked individuals went to the same general area used by the respective breeding population (S1 Fig.), justifying the pooling of data from all individuals in models. Based on the 50% and 90% density contours from kernel analysis, tracked birds from both populations were highly pelagic during their non-breeding period (Fig. 1). The area used by the Antipodes population was in the central south Pacific Ocean centred at 50°S and 115°W, and the area used by the Kerguelen population was in the south-east Indian Ocean centred at 45°S and 105°E. A southward shift in the monthly 50% density contours from October through February is apparent for both populations.

A total of 2604 and 1106 GLS locations fell within monthly 50% density contours for the Antipodes and Kerguelen populations, respectively; these were the presence locations used in the SDMs (Table 1). A total of 867 and 452 background locations were generated for each month for the Antipodes and Kerguelen models, respectively. In the combined model, presence and background locations from the Antipodes population were down-weighted by 0.76. Model validation (in terms of extrapolation) was conducted using 1180 filtered GLS locations from the four loggers that provided data from grey petrels at Marion Island (Table 1), of which 665 points fell within the 50% density contour (presence locations) that were compared with 435 background locations generated within the 90% density contour.

**Table 1:** Summary of geolocator (GLS) data obtained for the non-breeding period from grey petrels at Antipodes, Kerguelen and Marion islands, and used in distribution models and validation procedures.

Model	Month	Filtered GLS points	GLS locations in 50% contour considered presence	Presence locations withheld for validation^a^	Presence/Absence ratio in models
Antipodes					
	Oct	877	478	96	382/767
	Nov	1058	595	119	476/767
	Dec	1108	614	123	491/767
	Jan	1097	611	122	489/767
	Feb	661	306	61	245/767
	TOTAL	4801	2604	521	2083/3835
Kerguelen					
	Oct	230	133	27	106/352
	Nov	463	248	50	198/352
	Dec	527	296	59	237/352
	Jan	496	271	54	217/352
	Feb	287	158	32	126/352
	TOTAL	2003	1106	222	884/1760
Combined				743	2967/5595
Marion					
	Oct	229	128	---	---
	Nov	240	136	---	---
	Dec	243	137	---	---
	Jan	248	137	---	---
	Feb	220	127	---	---
	TOTAL	1180	665	---	---

^a^One hundred absence points were withheld from each month from both populations.

### SDM results and validation

Both population models performed well based on internal performance metrics (Antipodes: AUC = 0.96, dev = 0.62; Kerguelen: AUC = 0.91, dev = 0.45). Six of seven predictor variables were common between the Antipodes and Kerguelen models (Table 2). Month was not influential in either model and did not interact with other predictor variables, i.e., birds did not change their habitat use patterns between months. The combined model, based on the six common variables, also performed well based on internal performance metrics (AUC = 0.93, dev = 0.50).

**Table 2:** Boosted regression tree model parameters and results for the Antipodes and Kerguelen grey petrel populations, and combined model using data from both populations.

Model	Parameters (contribution)	# of interactions	Weight applied	Learning rate	Number of trees	dev	AUC
Antipodes	Depth (22.1)	4	None	0.015	5100	0.62	0.96
	MLD (21.0)						
	T0–50 (19.3)						
	Chl (15.4)						
	Curr (13.4)						
	EKE (8.8)						
Kerguelen	T0–50 (19.6)	4	None	0.0075	4900	0.45	0.91
	MLD (18.3)						
	EKE (16.7)						
	Curr (13.9)						
	Depth (13.7)						
	Chl (9.9)						
	Dist_sm (7.8)						
Combined	MLD (20.7)	4	Down-weighted Antipodes data by 0.76	0.0375	3250	0.5	0.93
	EKE (19.8)						
	T0–50 (17.9)						
	Chl (14.5)						
	Depth (14.2)						
	Curr (12.9)						

dev = Cross-validation per cent deviance explained; AUC = Area under the receiver operator curve; MLD = Mixed layer depth; T_0–50_ = Mean temperature between 0 and 50 m; Chl = Chlorophyll *a* concentration; Curr = Surface current velocity magnitude; EKE = Eddy kinetic energy; Dist_sm = Distance from seamount

Interpolation of the Antipodes and Kerguelen models within the same region resulted in high validation scores (Antipodes: r_s_ = 0.988 (*p* < 0.0001), COR = 0.811; Kerguelen: r_s_ = 0.962 (*p* < 0.0001), COR = 0.651). When the combined model in each region was interpolated, predictive performance was also high (to Antipodes: r_s_ = 0.971 (*p* < 0.0001), COR = 0.881; to Kerguelen: r_s_ = 0.955 (*p* < 0.0001), COR = 0.957). Furthermore, the calibration plots using withheld data from each model (interpolation) indicated that all three models were well calibrated (straight line from (0,0) to (1,1); S2 Fig.).

However, when the population models were extrapolated between regions, the models demonstrated poor predictive capacity and calibration (Antipodes model to Kerguelen: r_s_ = 0.024 (*p* = 0.949), COR = 0.029; Kerguelen model to Antipodes: r_s_ = -0.5711 (*p* = 0.085), COR = -0.003). Moreover, the models were poorly calibrated to the grey petrel distribution dataset from Marion Island: Antipodes model r_s_ = -0.186 (*p* = 0.607), COR = 0.062; Kerguelen model r_s_ = -0.361 (*p* = 0.306), COR = 0.106; combined model r_s_ = -0.663 (*p* = 0.037), COR = 0.008. The significant negative correlation between the combined model and the independent Marion data further demonstrates the limited transferability of this model to a novel region. Poor model calibration was also apparent in the calibration plots derived for all models when evaluated with data from another population (extrapolation; S2 Fig.). All models underestimated habitat suitability when the data were dominated by presences, indicating that the true habitat suitability was larger than the estimate given by the model. In contrast, the models overestimated habitat suitability when the data were dominated by background locations.

**Fig 2:**
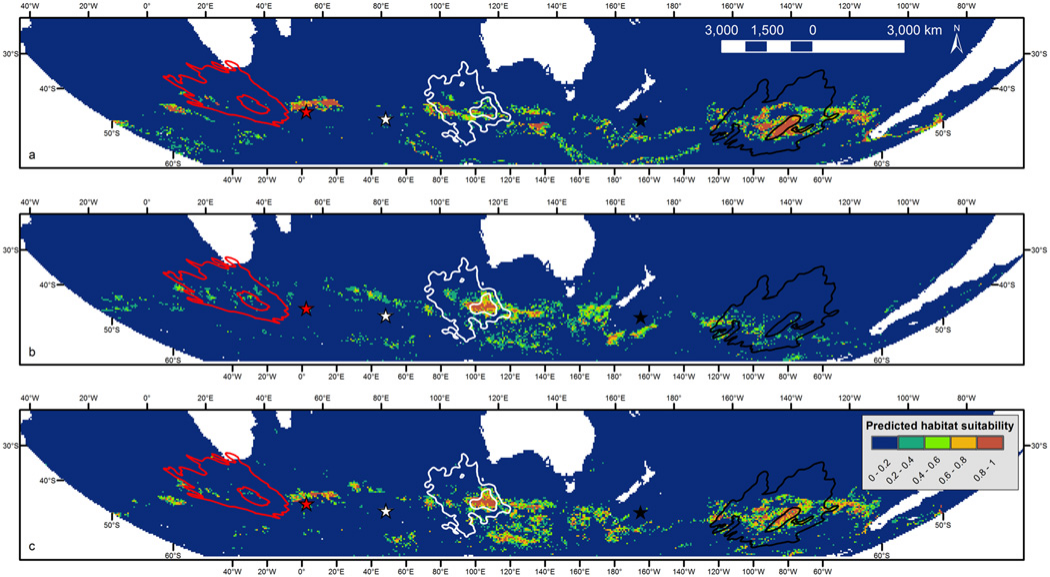
Predicted habitat suitability for grey petrels in January across the Southern Hemisphere. Predictions derived from boosted regression tree models for the (a) Antipodes, (b) Kerguelen and (c) combined populations. Location of grey petrel colonies at Antipodes Island (black star), Kerguelen Island (white star), and Marion Island (red star) shown. The January 50% and 90% density contours for tracked grey petrels from Antipodes (black lines), Kerguelen (white lines), and Marion (red lines) islands are displayed. Predicted habitat suitability ranges from low (0) to high (1) on a constant colour scheme between plots. Maps in Molleweide, datum wgs84.

Results of the model validations are reflected in the spatial predictions of habitat suitability for grey petrels across the Southern Hemisphere by each model for each month (January displayed in Fig. 2; other months in S3 A-E Fig.). All three models demonstrated high interpolative capability. However, when the models were transferred to novel regions to test their ability to extrapolate spatial predictions, very low habitat suitability was incorrectly predicted for areas of known occurrence of this species (*e*.*g*., within the 50% density contours for tracked birds).

### Ecological relationships derived by SDMs

**Fig 3:**
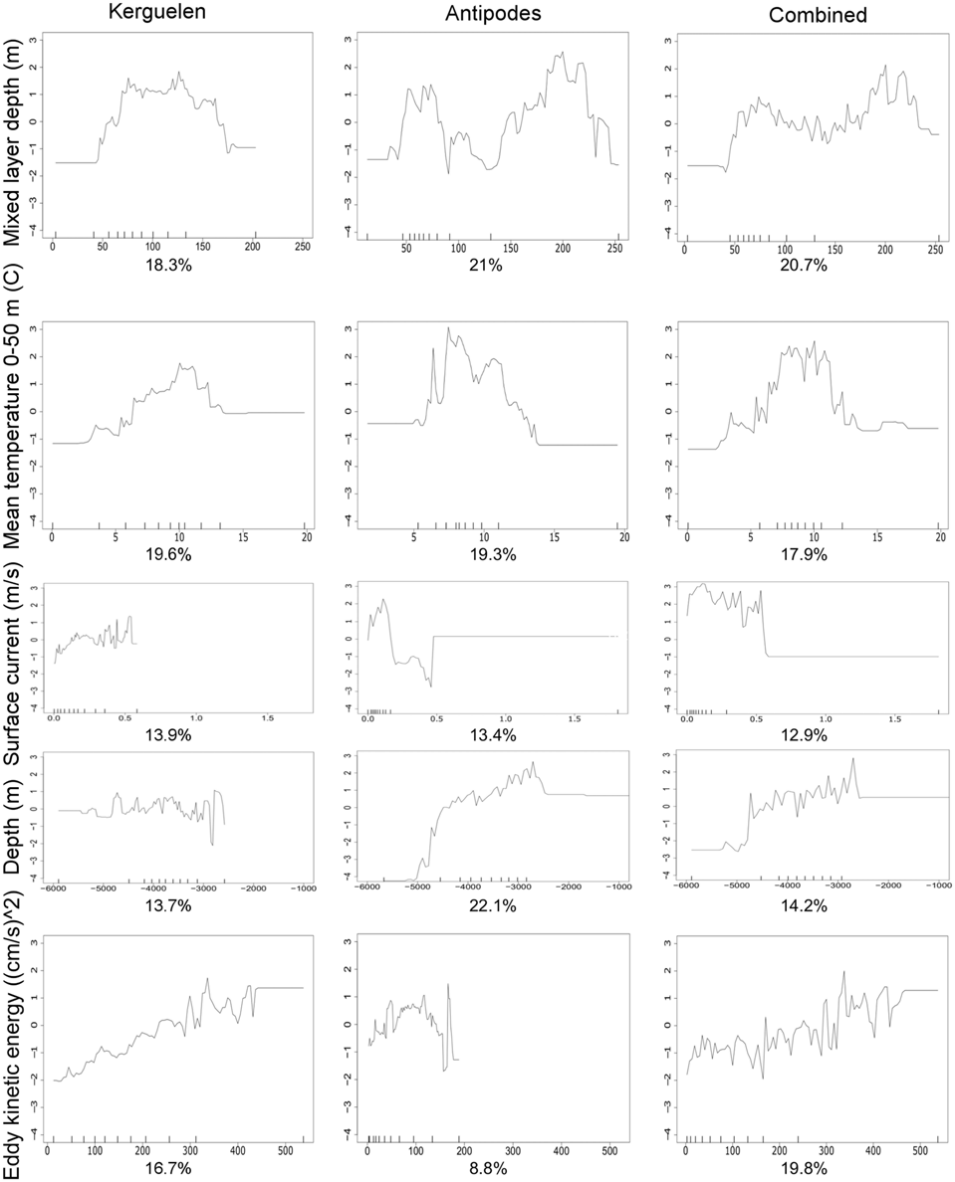
Fitted functions between grey petrel distribution and the five most influential predictor variables. Functional relationships determined by boosted regression tree models for the Kerguelen (1st column), Antipodes (2nd column), and combined (3rd column) populations. Plots indicate the marginal effect on petrel use/availability (y-axes) by each predictor variable (x-axis). Contribution of each variable to the model given below the function. Y-axes are on a logit scale and are common across all plots. Scale of x-axes is common across each predictor variable. Rug plots show distribution of data across that variable, in deciles, and are used as a measure of confidence in the shape of the fitted-function.

**Fig 4:**
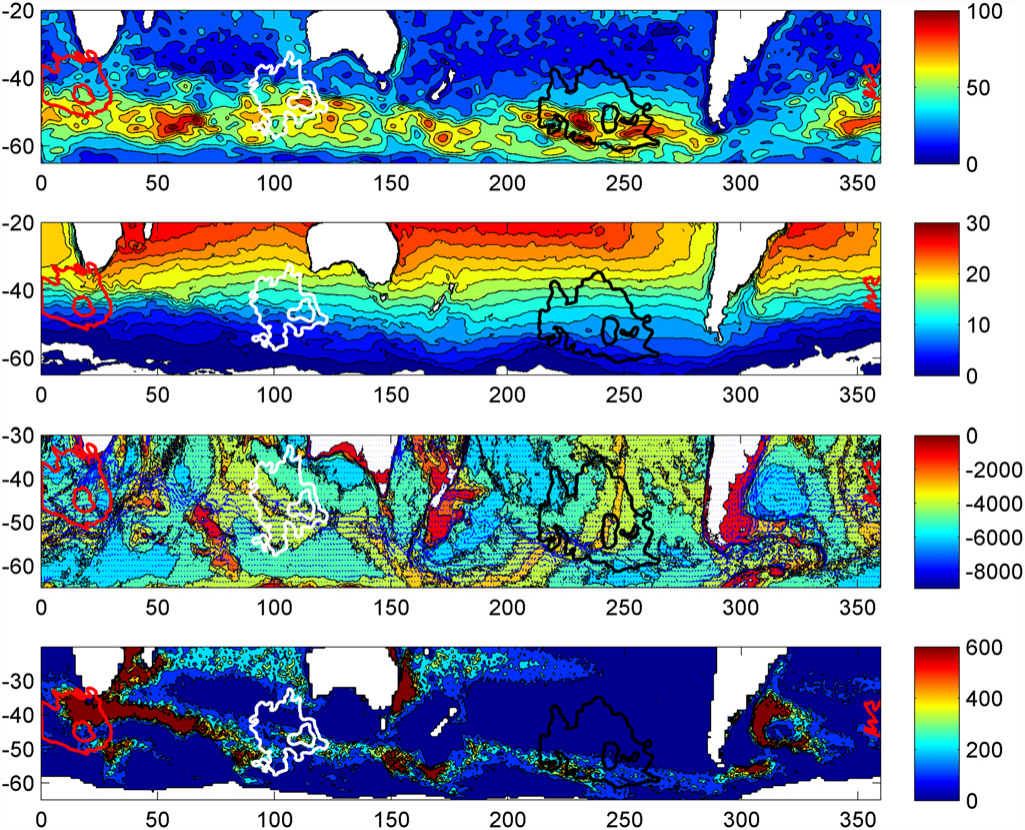
Grey petrel distribution in January from three colonies overlaid on January oceanographic climatologies. The January 50% and 90% density contours for tracked grey petrels from Antipodes (black lines), Kerguelen (white lines), and Marion (red lines) islands are displayed. (a) Mixed layer depth (m), (b) mean temperature in upper 50m (C°), (c) surface currents (m/s) over depth (m), (d) eddy kinetic energy ((cm s-1)^2^). Maps in native projection of environmental layers: geographic, datum wgs84.

Although the population models indicated similar ecological relationships between grey petrel distribution data and the common predictor variables, the shape of these functions varied relative to the range of environmental variation (Fig. 3; note that portions of functional relationships with little data as indicated by rug plots are disregarded during interpretation). Mixed layer depth (MLD) had a large contribution in all three models (21–18%; Table 2), with the results indicating that grey petrels used habitat with a similar range of MLDs (Antipodes: 50–80 m; Kerguelen: 50–130 m; Combined: 50–100 m). Yet, the distribution of presences (50% contour) and available habitat (90% contour) relative to composite images of MLD in January (Fig. 4a) indicate that tracked birds had variable selection patterns in relation to available habitat: the Kerguelen birds used habitat with the deepest available MLD, whereas birds from Antipodes used habitat over a ridge of shallow MLD between two deeper areas, and those from Marion used an area with moderate MLD adjacent to an area with shallow MLD. These results illustrate how inference of species habitat preference via use-availability models is dependent on the available habitat assessed [47].

The mean temperature between the surface and 50 m (t_0–50_) contributed 18–19% to all three SDMs, and the fitted functions indicated a preference for habitat with temperatures between 7 and 13°C in the upper 50 m (Fig. 3). Comparison of grey petrel distribution in January from all three tracked populations with a composite image of t_0–50_ shows that birds selected habitat between the Subtropical and Sub-Antarctic fronts (Fig. 4b). Surface current velocity (curr) contributed 13–14% to all models; all relationships indicated an increase in petrel presence between current velocities of 0–0.2 m.s^-1^, and a decrease in habitat suitability with strong currents above 0.2 m.s^-1^ (Fig. 3). Indeed, grey petrels appear to select against available habitats with the strongest currents, and concentrate in adjacent areas with moderate current velocities (Fig. 4c). Birds from Antipodes Island were distributed east of the swift Antarctic Circumpolar Current (ACC); grey petrels from Kerguelen used habitat just to the north of the ACC; and those from Marion Island used an area south of the dynamic Agulhas Retroflection and north of the ACC. This pattern is also reflected in the relationships with eddy kinetic energy (EKE). None of the grey petrel populations selected habitats with high EKE; birds from Antipodes and Marion Island used areas with low EKE, and those from Kerguelen used an area with moderate EKE (Figs. 3 and 4d).

Although depth was an important contributor to all three models (Antipodes: 22%; Kerguelen: 14%; Combined: 14%), the relationships between depth and distribution differed for grey petrels from Antipodes and Kerguelen (Fig. 3). Petrels from Antipodes demonstrated a linear increase in presence with decreasing depth from 5000 to 3000 m, and selected habitat along the East Pacific Rise (Fig. 4c). In comparison, the grey petrels from Kerguelen had no clear relationship with depth, and used habitats with depths of 3500–4500 m just to the north of the Southeast Indian Ridge. Both the East Pacific Rise and the Southeast Indian Ridge strongly influence current location and velocity in these respective regions [48]. However, the Marion Island birds used deep water habitats (~5000 m) and were not spatially proximate to bathymetric features affecting regional currents.

## Discussion

At the broad scale of the Southern Hemisphere, grey petrels from Antipodes and Kerguelen islands appear to use non-breeding habitat with the same general characteristics: near oceanic ridges in areas with moderate current velocities, where the mixed layer depth is 50–100 m, and just north of the Sub-Antarctic Front with a mean temperature in the upper 50 m of 7–13°C. However, the precise patterns of habitat use by birds from each population differed substantially. Subsequently, predictive performance of the three SDMs when interpolated within the calibration regions was high, but extrapolation tests demonstrated low transferability to novel regions. Despite aggregating data from the two populations into a theoretically more generalized SDM for grey petrels, the combined model also performed poorly when extrapolated to the Marion Island dataset. We conclude that grey petrels share potential distribution patterns relative to large-scale environmental variation, but differences in habitat availability and climate across the regions used by non-breeding grey petrels from the different colonies creates variable realized distribution patterns for each population. This ratio of habitat use to availability is non-linear and ultimately drives the coefficients and shape of model derived resource selection functions [49,50]. Therefore, functional response in resource selection should not be ignored when model results are applied to conservation efforts, such as predictions of realized habitat [51].

Model parameters can cause poor performance, such as an inappropriate choice of modelling method or scale of analysis, and inclusion of ecologically irrelevant, or spatially or temporally inaccurate predictor variables. However, we believe it is unlikely that poor model extrapolation in this study is due to methodological choices because (a) BRTs are an appropriate SDM method for interpreting complex species-environment relationships [30,42], (b) grain size is not expected to affect SDM predictions generated by BRTs [52], and (c) the models generated ecologically intuitive and broadly consistent relationships of habitat use by each population, and produced high validation scores when interpolated. However, poor model extrapolations can occur if all multivariate combinations of the environmental conditions assessed in the training dataset are not represented in the novel dataset [53]. This scenario is possible in our study where models were transferred across large ocean basins. Such overfitting of models should be avoided to enhance predictive capacity by balancing model applicability with model complexity [54]. If poor model extrapolation was due to overfit models in our study, we would expect the combined model to show greater predictive capacity than the single population models due to a more generalized description of grey petrel distribution patterns that accounted for regional differences in oceanographic and habitat use patterns. Hence, more plausible explanations for poor model transferability include ecological factors, such as variability in grey petrel diet preference or another aspect of behaviour (e.g., degree of fisheries interactions), or different combinations of oceanographic patterns that affect prey concentration and availability to the grey petrels in the different ocean regions across the Southern Hemisphere.

Unlike previous studies that used data from multiple regions to develop generalized models with high transferability between sites up to 53 km apart [4,55], our extrapolation of the combined model failed over much greater distances due to regional species-environment relationships not captured in the model. Nevertheless, assessment of the resource selection functions derived by the three SDMs enables a comparison of realized versus potential distributions to enhance our understanding of large-scale habitat use by grey petrels across the Southern Hemisphere. SDMs for grey petrels from the Antipodes and Kerguelen populations included a similar suite of influential predictor variables, but realized distributions differed relative to habitat availability in each ocean basin. For instance, grey petrels were associated with a similar range of mixed layer depths that may enhance prey availability for shallow diving seabirds like grey petrels, but the distribution of birds relative to mixed layer depth availability indicates that tracked birds had variable resource selection patterns. In contrast, according to the SDMs, the functional relationships between petrel distribution and depth varied between populations; this was also evident for Marion Island birds, which used deeper water than the other populations. Bathymetry strongly influences the Antarctic Circumpolar Current (ACC) across the range of grey petrels in the Southern Hemisphere by steering the flow around ridges and through fracture zones [48]. The ACC demonstrates unidirectional flow from top to bottom so that deep features, such as the East Pacific Rise and Southeast Indian Ridge, determine surface flow direction and speed, and horizontal density gradients [48], and hence prey availability to predators [16].

Linking marine predator distributions to oceanography is typically reliant on good proxy relationships between available environmental data and unavailable prey distribution data e.g., [56,57]. Our study is no exception, yet we have progressed our understanding of the links between environment, prey and marine predators in two ways. First, the distributions of the non-breeding grey petrels from all three islands were entirely unexpected as there was little prior evidence that these regions were important seabird foraging grounds. Tracking data have not been collected for the majority of procellariiforms [58], making it likely that other seabirds use these areas during the summer months. Second, although the exact mechanism of increased prey availability to grey petrels in their non-breeding areas remains unknown [59], we documented large-scale similarities in oceanographic patterns that likely facilitate foraging opportunities. Due to the extended residency periods (4–5 months) of the grey petrels in these pelagic areas, further research is warranted, ideally including *in situ* observations and prey sampling.

The conservation implications of these results are apparent when non-breeding distributions of birds from the three populations and SDM predictions are compared to fishing effort. An analysis of the spatial distribution of reported pelagic longline fishing effort south of 30°S between 1960 and 1998 indicates that the region used by the grey petrels from Kerguelen Island incurs intense fishing effort, whereas little fishing effort was reported for the region used by non-breeding grey petrels from Antipodes [60]. This contrasting level of overlap with fishing effort may contribute to the divergent population trends, as the Antipodes population is believed to be stable [61] and the Kerguelen Island population may be in decline due to low adult survival [25]. SDMs based on data for the Antipodes and Kerguelen populations incorrectly predict the main non-breeding habitat of grey petrels from Marion Island to be in an area of low fishing effort. In contrast, the tracking data from that site indicate that grey petrels from Marion overlap with high levels of longling fishing effort. High at-sea mortality of this population is consistent with its failure to recover since feral cats *Felis catus* were removed from the island in 1990 [62]. In this case, the false SDM prediction could have led to misinformed management decisions regarding requirements for fisheries bycatch mitigation.

Predictive SDMs are increasingly being touted as a useful conservation tool, including for marine predators such as pinnipeds, cetaceans and seabirds [16,34], despite few tests of model transferability to novel areas. This study used SDMs to explain and predict habitat use by grey petrels in different ocean basins across the Southern Hemisphere. We extrapolated models between regions nearly 10,000 km apart, which is a more rigorous test of transferability than previous studies [4,5,12,23,42]. Strong interpolation and weak extrapolation indicate that our models are able to describe the potential response of grey petrels to environmental variation, but the realized response of each grey petrel population to oceanographic conditions is context-dependent. Given the high conservation concern and wide geographic ranges of many marine predators, it is tempting to apply SDMs to prioritize protected areas and inform conservation management. However, we recommend caution when attempting to extrapolate model results outside the calibration region. Although SDMs can elucidate a species’ potential distribution pattern relative to large-scale environmental conditions, data on local habitat availability and preference is necessary to understand and successfully predict the realized distribution of a population in a different region. We advocate increased testing of SDM transferability across large and ecologically-relevant scales to improve extrapolation capability and better inform conservation applications.

## Supporting Information

S1 Fig Filtered, raw GLS locations from tagged grey petrels from Antipodes and Kerguelen islands. (PDF)

S2 Fig Calibration plots of the Antipodes, Kerguelen and combined models derived from interpolation and extrapolation tests. (PDF)

S3 Fig Predicted monthly non-breeding habitat suitability for grey petrels across the Southern Hemisphere. (PDF)
