# Supplementary material for: Poor Transferability of Species Distribution Models for a Pelagic Predator, the Grey Petrel, Indicates Contrasting Habitat Preferences across Ocean Basins
Source: PLoS One. 2015 Mar 6;10(3):e0120014. doi: 10.1371/journal.pone.0120014 (PMC4352036; doi:10.1371/journal.pone.0120014)
Supplement: S1 Fig — (PDF) [file pone.0120014.s001.pdf]

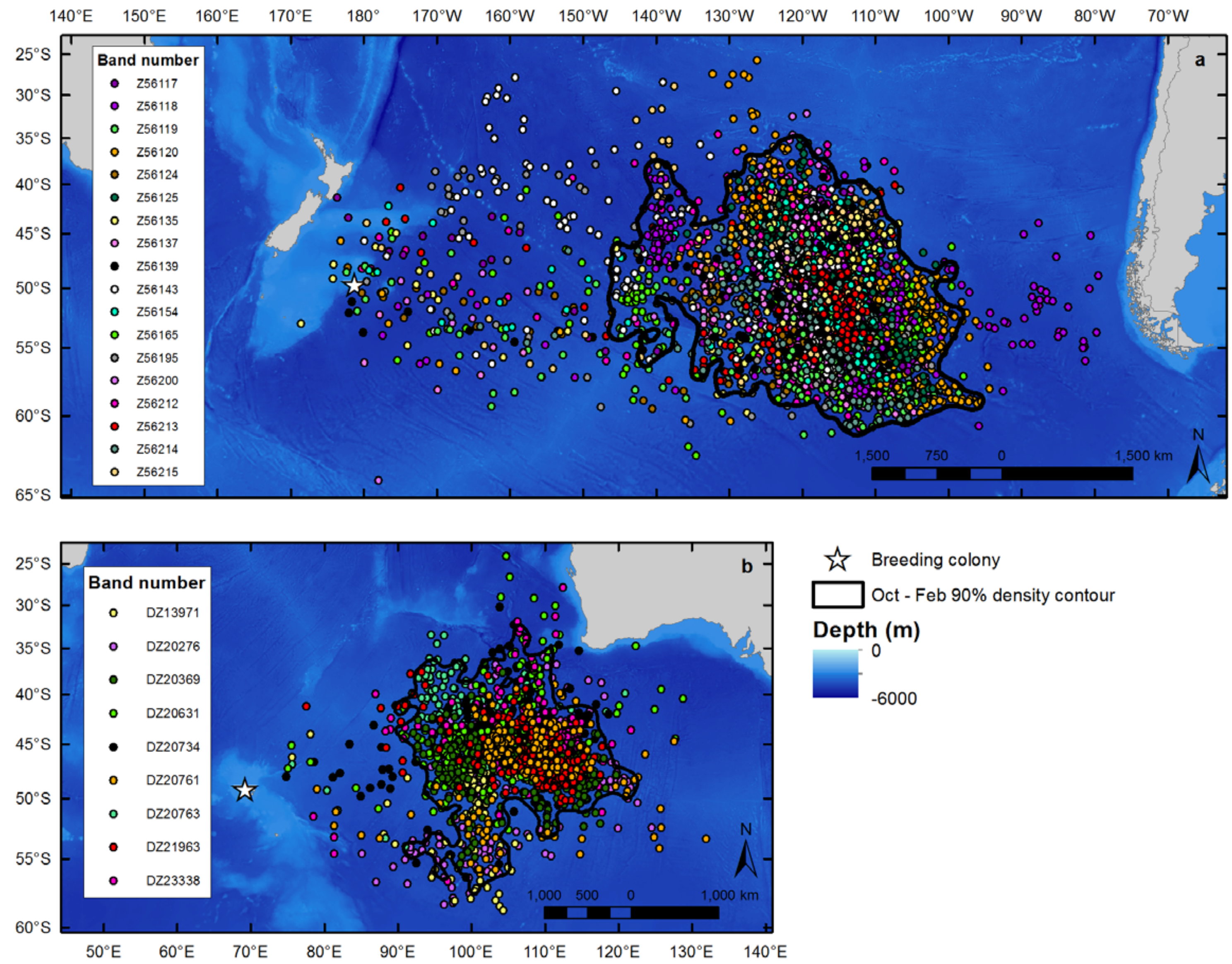


S1 Figure. Filtered, raw GLS location from individual grey petrels tagged at (a) Antipodes Island (n=18) and (b) Kerguelen Island (n= 9), which were implemented in the boosted regression tree models of species distribution. Regional bathymetry and the 90% density contour of GLS locations between October and February from each breeding colony are shown for reference.
