# Supplementary material for: Poor Transferability of Species Distribution Models for a Pelagic Predator, the Grey Petrel, Indicates Contrasting Habitat Preferences across Ocean Basins
Source: PLoS One. 2015 Mar 6;10(3):e0120014. doi: 10.1371/journal.pone.0120014 (PMC4352036; doi:10.1371/journal.pone.0120014)
Supplement: S2 Fig — (PDF) [file pone.0120014.s002.pdf]

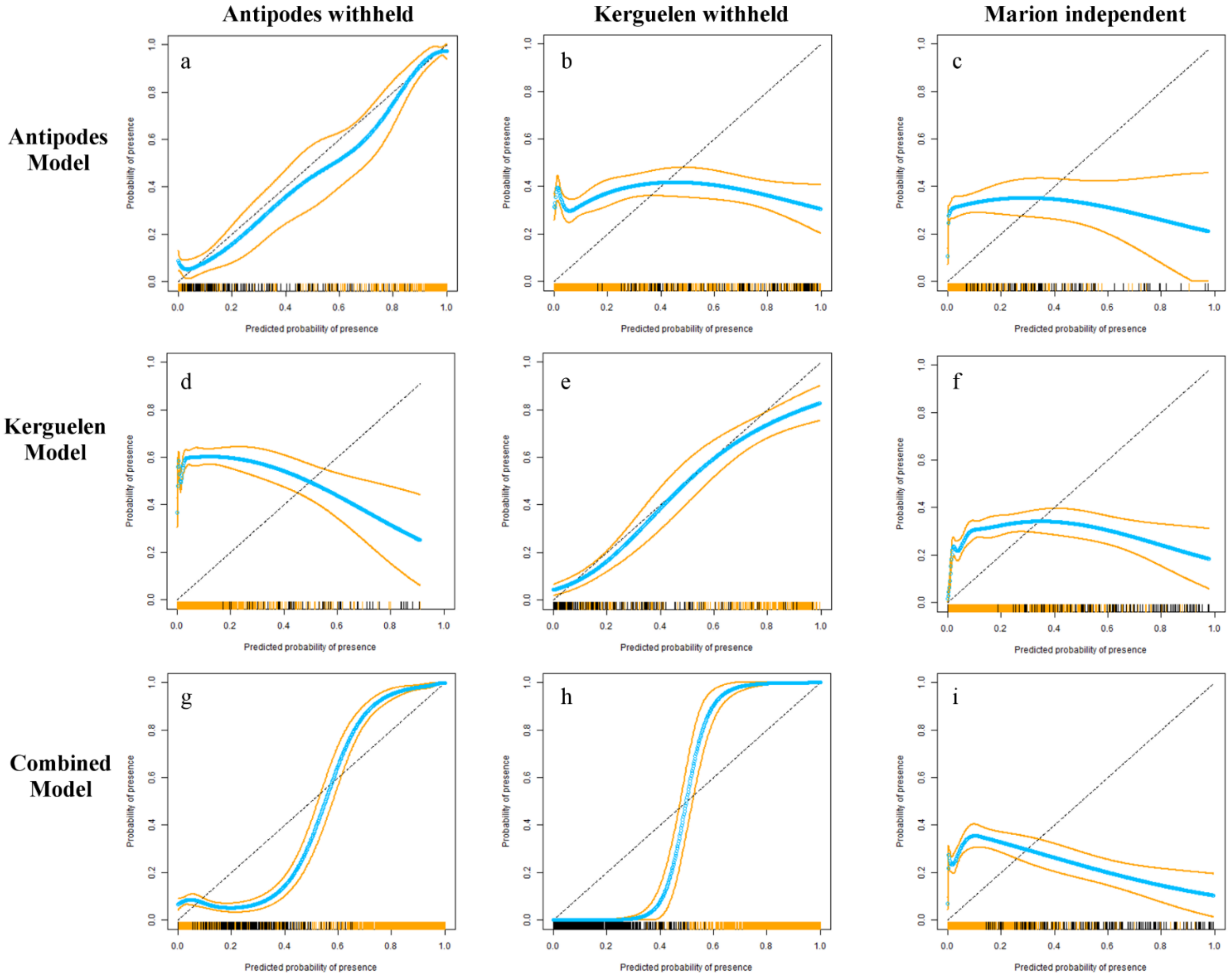

S2 Figure. Presence–absence calibration plots for species distribution models generated for grey petrels from Antipodes Island (a-c), Kerguelen Island (d-f), and a combined model from both Antipodes and Kerguelen islands (g-i). Models were calibrated using withheld data from the Antipodes population (a,d,g), Kerguelen population (b,e,h), or the independent tracking data from Marion Island (c,f,i). Shown are the calibration curves (cyan) and a confidence interval of ±2 SD (orange). The rug plots show model values at presence (orange) and absence (black) points. Plots made using code provided by (Phillips & Elith, 2010).

References:

Phillips, S.J. & Elith, J. (2010) POC plots: calibrating species distribution models with presence-only data. *Ecology*, **91**, 2476-2484.
