# Supplementary material for: Poor Transferability of Species Distribution Models for a Pelagic Predator, the Grey Petrel, Indicates Contrasting Habitat Preferences across Ocean Basins
Source: PLoS One. 2015 Mar 6;10(3):e0120014. doi: 10.1371/journal.pone.0120014 (PMC4352036; doi:10.1371/journal.pone.0120014)
Supplement: S3 Fig — (PDF) [file pone.0120014.s003.pdf]

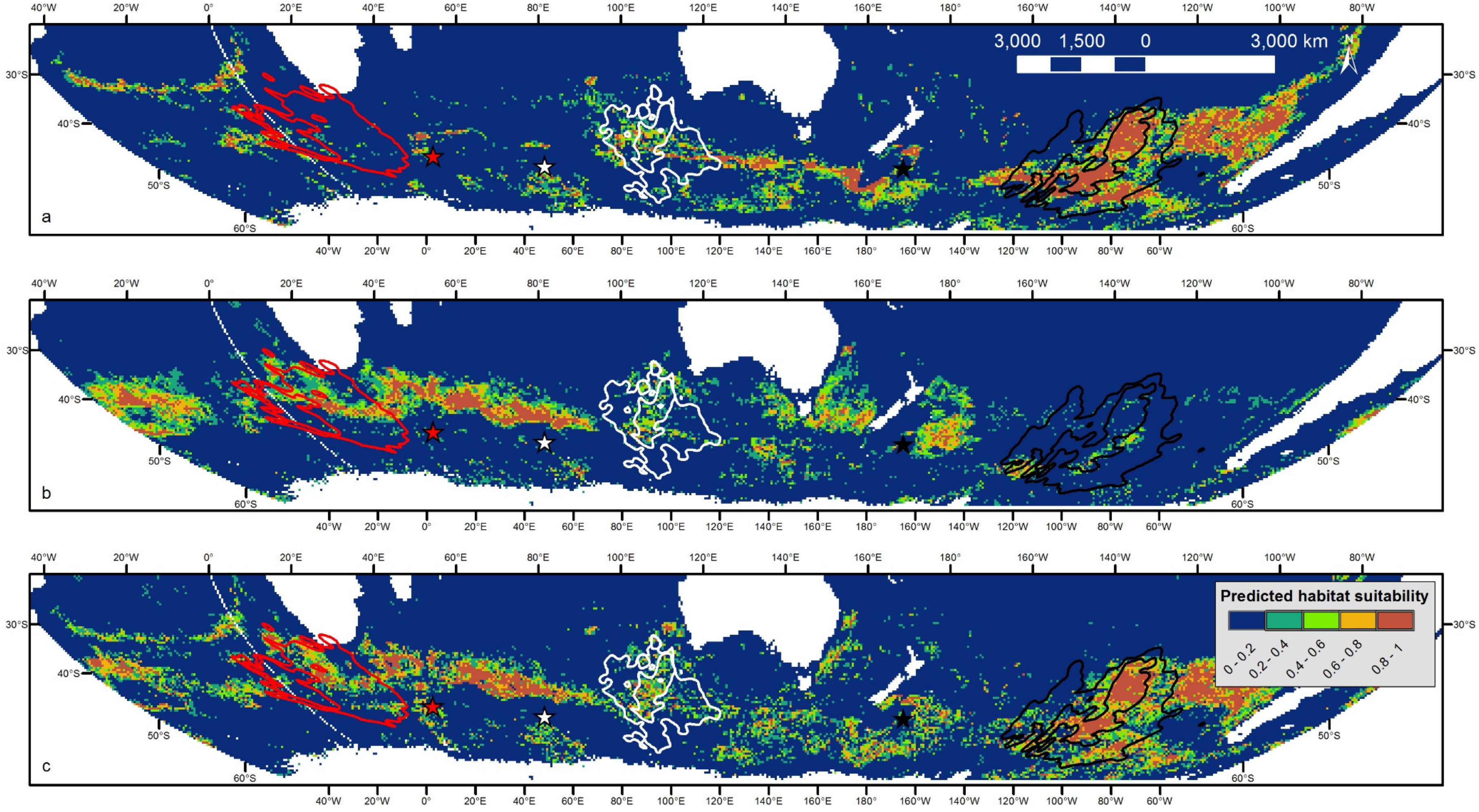

Figure A. Predicted habitat suitability for grey petrels during October across the Southern Hemisphere, derived from boosted regression tree models for the (a) Antipodes, (b) Kerguelen and (c) combined populations. Location of grey petrel colonies at Antipodes Island (black star), Kerguelen Island (white star), and Marion Island (red star) shown. The October 50% and 90% density contours for tracked grey petrels from Antipodes (black lines), Kerguelen (white lines), and Marion (red lines) islands are displayed. Predicted habitat suitability ranges from low (0) to high (1) on a constant colour scheme between plots. Maps in Molleweide, datum wgs84.

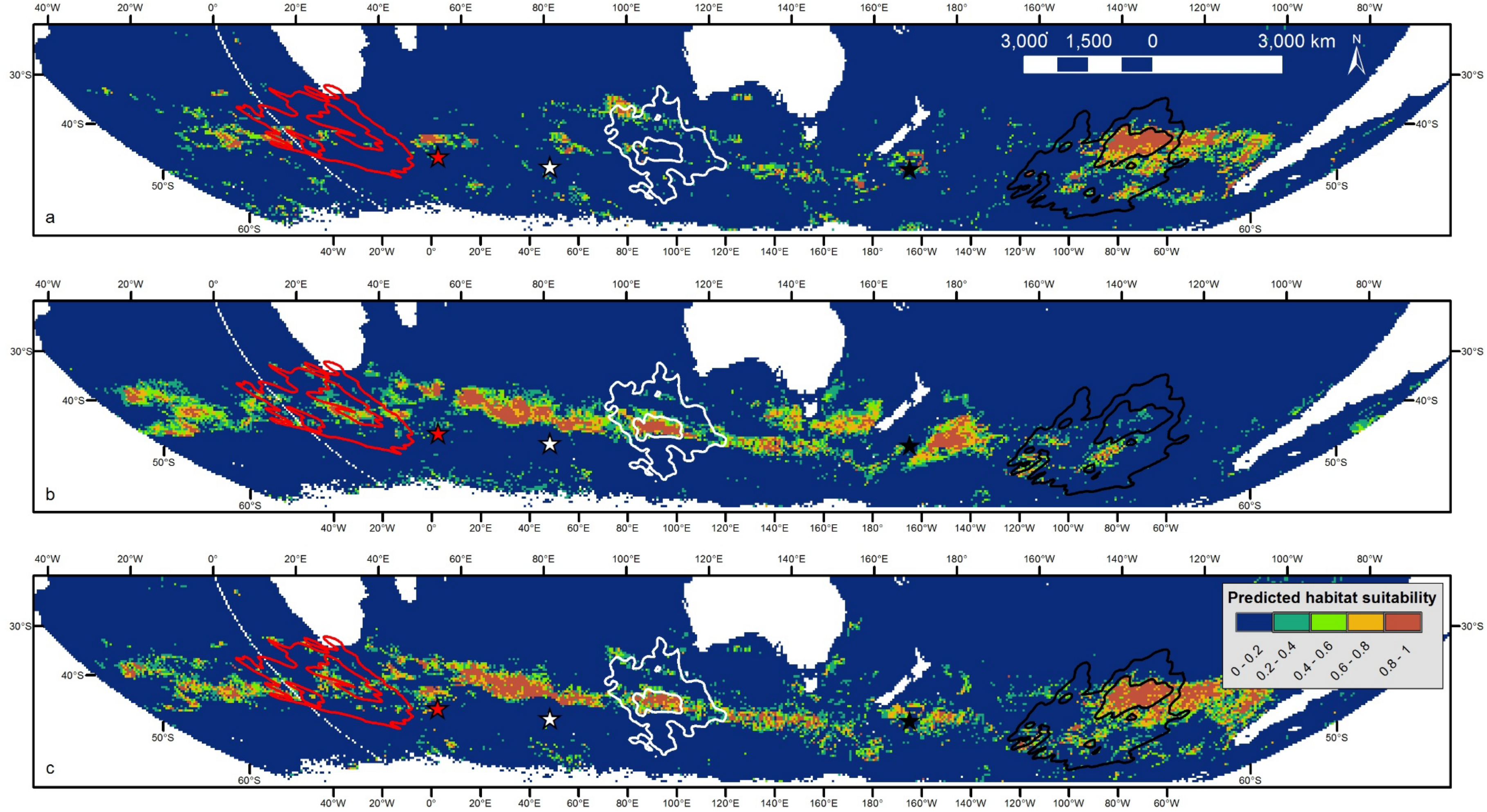

Figure B. Predicted habitat suitability for grey petrels during November across the Southern Hemisphere, derived from boosted regression tree models for the (a) Antipodes, (b) Kerguelen and (c) combined populations. Location of grey petrel colonies at Antipodes Island (black star), Kerguelen Island (white star), and Marion Island (red star) shown. The November 50% and 90% density contours for tracked grey petrels from Antipodes (black lines), Kerguelen (white lines), and Marion (red lines) islands are displayed. Predicted habitat suitability ranges from low (0) to high (1) on a constant colour scheme between plots. Maps in Molleweide, datum wgs84.

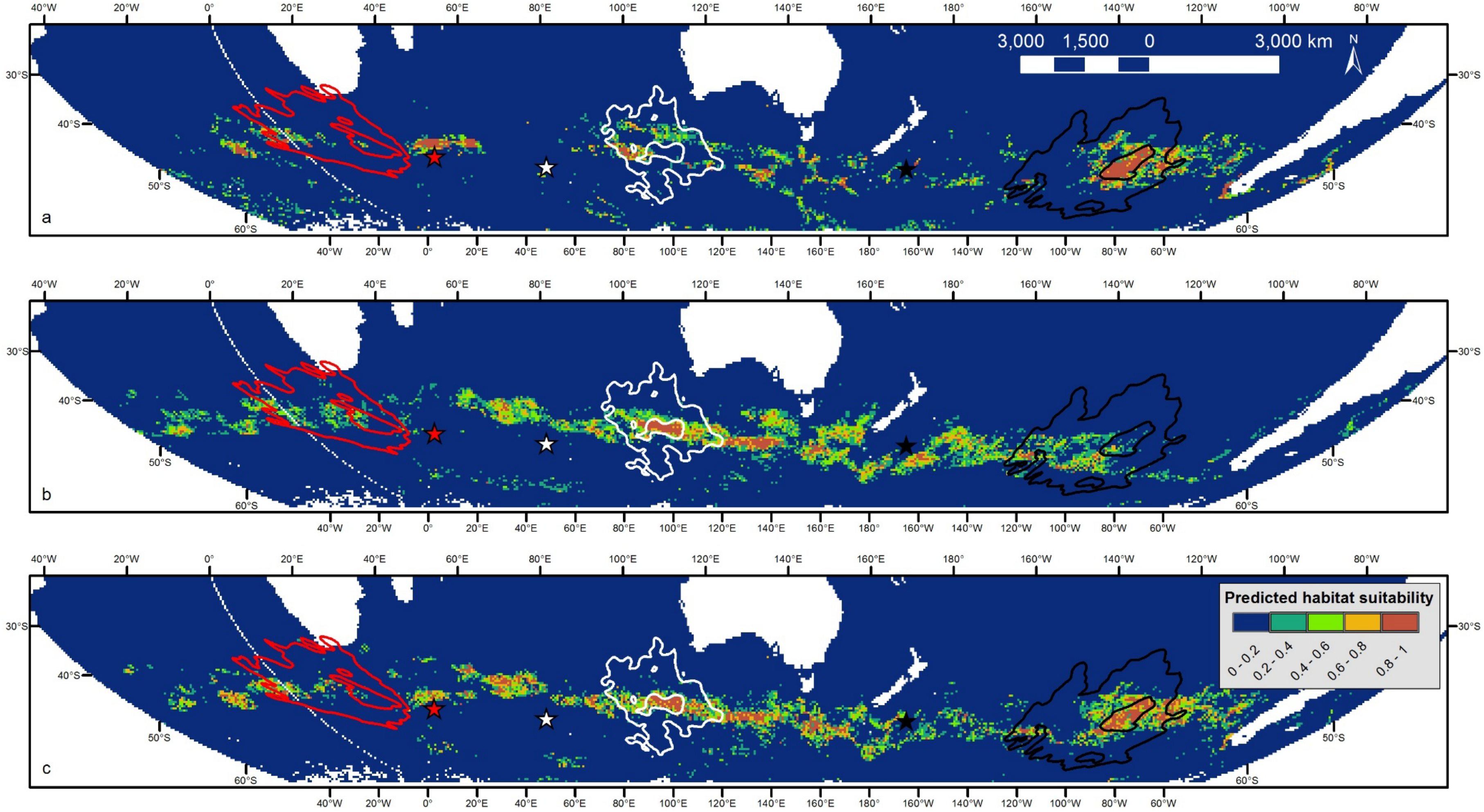

Figure C. Predicted habitat suitability for grey petrels during December across the Southern Hemisphere, derived from boosted regression tree models for the (a) Antipodes, (b) Kerguelen and (c) combined populations. Location of grey petrel colonies at Antipodes Island (black star), Kerguelen Island (white star), and Marion Island (red star) shown. The December 50% and 90% density contours for tracked grey petrels from Antipodes (black lines), Kerguelen (white lines), and Marion (red lines) islands are displayed. Predicted habitat suitability ranges from low (0) to high (1) on a constant colour scheme between plots. Maps in Molleweide, datum wgs84.

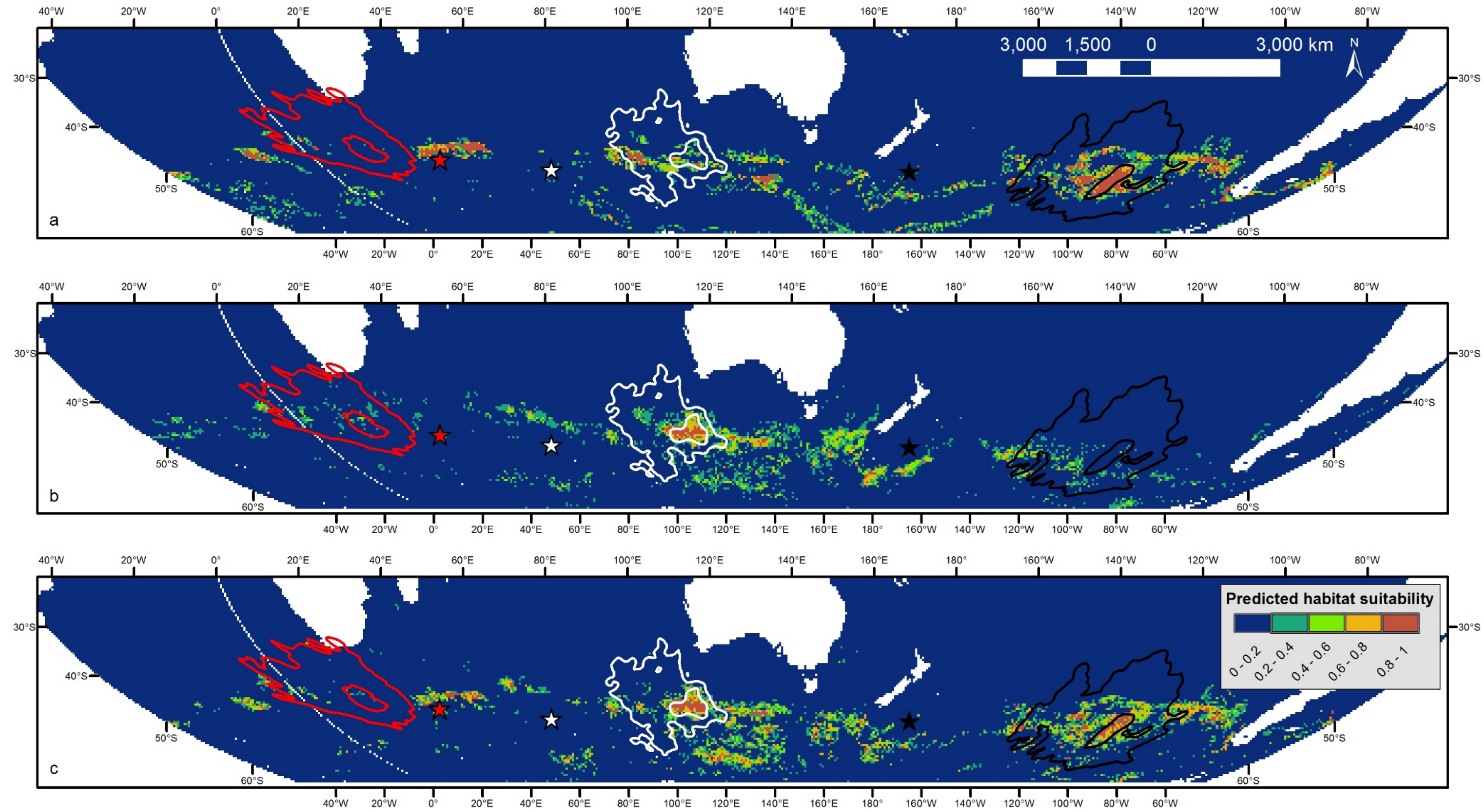

Figure D. Predicted habitat suitability for grey petrels during January across the Southern Hemisphere, derived from boosted regression tree models for the (a) Antipodes, (b) Kerguelen and (c) combined populations. Location of grey petrel colonies at Antipodes Island (black star), Kerguelen Island (white star), and Marion Island (red star) shown. The January 50% and 90% density contours for tracked grey petrels from Antipodes (black lines), Kerguelen (white lines), and Marion (red lines) islands are displayed. Predicted habitat suitability ranges from low (0) to high (1) on a constant colour scheme between plots. Maps in Molleweide, datum wgs84.

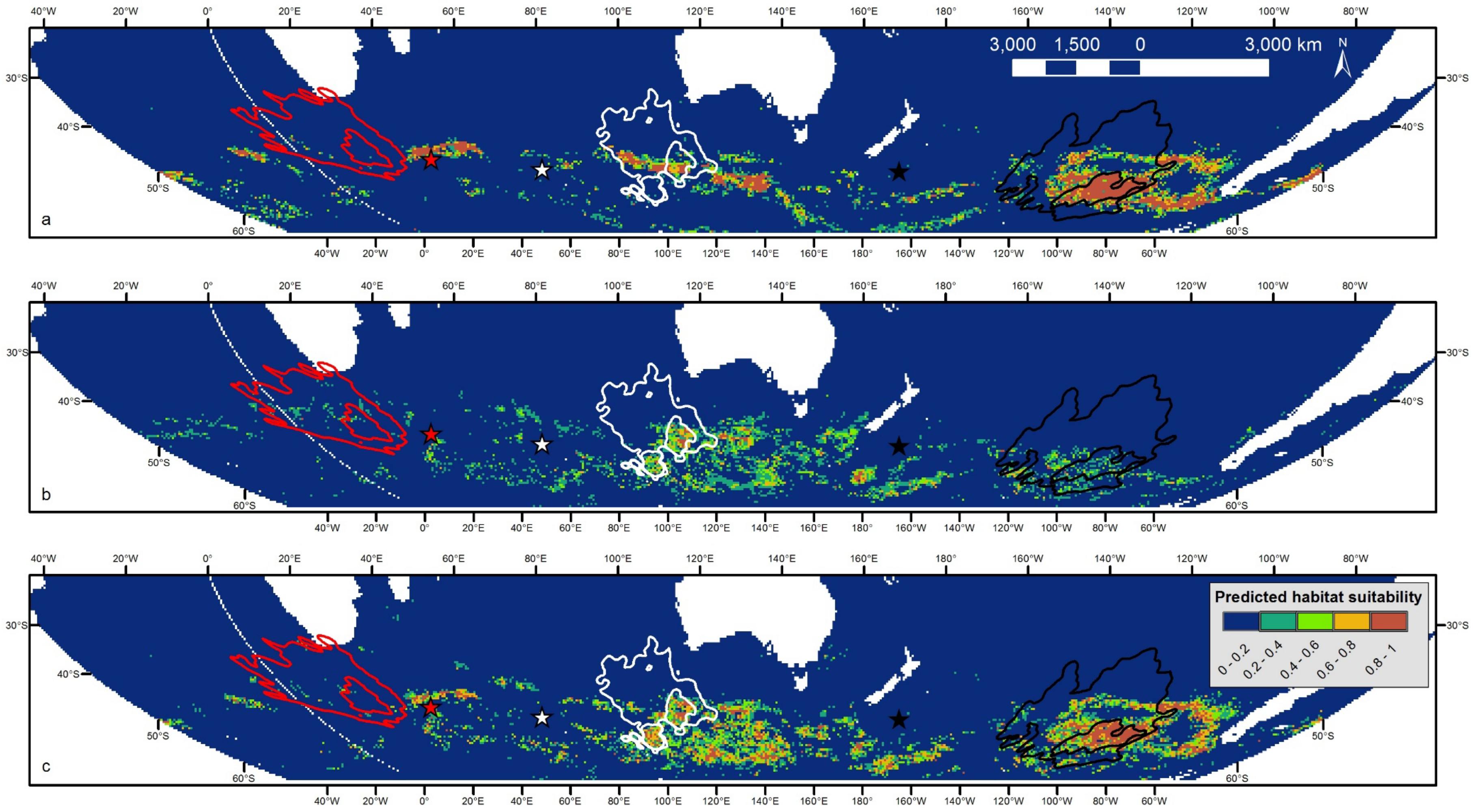

Figure E. Predicted habitat suitability for grey petrels during February across the Southern Hemisphere, derived from boosted regression tree models for the (a) Antipodes, (b) Kerguelen and (c) combined populations. Location of grey petrel colonies at Antipodes Island (black star), Kerguelen Island (white star), and Marion Island (red star) shown. The February 50% and 90% density contours for tracked grey petrels from Antipodes (black lines), Kerguelen (white lines), and Marion (red lines) islands are displayed. Predicted habitat suitability ranges from low (0) to high (1) on a constant colour scheme between plots. Maps in Molleweide, datum wgs84.
